# Dynamics of the Glycogen β-Particle Number in Rat Hepatocytes during Glucose Refeeding

**DOI:** 10.3390/ijms23169263

**Published:** 2022-08-17

**Authors:** Natalia N. Bezborodkina, Andrei V. Stepanov, Mikhail L. Vorobev, Grigory I. Stein, Sergey V. Okovityi, Boris N. Kudryavtsev

**Affiliations:** 1 Zoological Institute, Russian Academy of Sciences, Universitetskaya Emb, 1, 199034 St. Petersburg, Russia; 2 Group of Neuroregulation of Muscle Function, Sechenov Institute of Evolutionary Physiology and Biochemistry, Russian Academy of Sciences, Thorez Ave., 44, 194223 St. Petersburg, Russia; 3 Confocal Microscopy and Image Analysis Group, Institute of Cytology, Russian Academy of Sciences, Tikhoretsky Ave. 4, 194064 St. Petersburg, Russia; 4 Department of Pharmacology and Clinical Pharmacology, Saint Petersburg State Chemical Pharmaceutical University, 197022 St. Petersburg, Russia; 5 Department of Research Institute of Cardiovascular Diseases, Pavlov First Saint Petersburg State Medical University, L’va Tolstogo Str., 6-8, 197022 St. Petersburg, Russia

**Keywords:** glycogen, β-particles, rat hepatocytes, glucose, refeeding

## Abstract

Glycogen is an easily accessible source of energy for various processes. In hepatocytes, it can be found in the form of individual molecules (β-particles) and their agglomerates (α-particles). The glycogen content in hepatocytes depends on the physiological state and can vary due to the size and number of the particles. Using biochemical, cytofluorometric, interferometric and morphometric methods, the number of β-particles in rat hepatocytes was determined after 48 h of fasting at different time intervals after glucose refeeding. It has been shown that after starvation, hepatocytes contain ~1.6 × 10^8^ β-particles. During refeeding, their number of hepatocytes gradually increases and reaches a maximum (~5.9 × 10^8^) at 45 min after glucose administration, but then quickly decreases. The data obtained suggest that in cells there is a continuous synthesis and degradation of particles, and at different stages of life, one or another process predominates. It has been suggested that in the course of glycogenesis, pre-existing β-particles are replaced by those formed de novo. The main contribution to the deposition of glycogen is made by an increase in the glucose residue number in its molecules. The average diameter of β-particles of glycogen during glycogenesis increases from ~11 nm to 21 nm.

## 1. Introduction

Glucose is a widespread carbohydrate in nature. Glucose owes its appearance on our planet to oxygenic photosynthesis, the era of which began more than 2 billion years ago in early prokaryotes capable of fixing CO_2_ [1,2,3]. In the absence of glucose, the normal vital activity of the vast majority of organisms is apparently impossible, and therefore they must always have a certain supply of this carbohydrate. The reserve of glucose in animals is its polymeric form—glycogen. Glycogen serves as an easily accessible and universal source of energy for metabolic processes in living beings with different levels of organization, from primitive heterotrophic prokaryotes (archaea and bacteria) to higher eukaryotes (mammals) [4].

Glycogen is present in many mammalian tissues, but its concentration is especially high in the liver. Although the liver performs a huge number of different functions necessary for living abilities of the body, the most important of them is the glucostatic function. The role of this function in the body is to maintain a constant level of glucose in the blood. The key mechanism that provides the glucostatic function by the liver is the ability of its parenchymal cells (hepatocytes) to synthesize glycogen from blood glucose, as well as from non-carbohydrate substances, and break it down to glucose in response to the organism demands.

In the cytoplasm of hepatocytes, glycogen is stored in the form of special granules- β-particles (glycogen molecules). Often, agglomerates of 20–40 β-particles, connected by covalent bonds, form α-particles, the diameter of which can reach 200–300 nm [5,6]. The synthesis of glycogen, like other polysaccharides, is not carried out on a matrix, and therefore, unlike, for example, proteins, it is not strictly organized. The glycogen molecule does not have a constant composition due to the continuous circulation of glucose residues, as well as the endless attachment–detachment of enzymes involved in its synthesis and degradation. Nevertheless, the general plan of the glycogen molecule structure, the composition of its spatial structure, can be traced quite clearly and in the best possible way is suitable for the liver to perform its glucostatic function [7,8].

It is believed that a completely formed glycogen macromolecule (β-particle) has a diameter of 42–44 nm and a molecular weight of ~10^7^ Da and includes ~55,000 glucose residues connected by α-(1→4) and α-(1→6) glycosidic bonds arranged in 12 tiers [9,10,11,12]. In addition to the polysaccharide part, in the center of the β-particle there is glycogenin, a self-glycosylating protein, and on the glycogen molecule surface there are proteins that are directly involved in the attachment–detachment of glucose residues or in the regulation of these processes [13,14,15,16,17,18,19]. All these proteins are an integral part of the spatial structure of β-particles and together can make up to 80% of its mass [20]. An increase or decrease in the number of glucose residues in β-particles is accompanied by an increase or decrease in particle size, respectively. Thus, the content of glycogen in cells can change both due to the size of β-particles and their number. Only one work is known devoted to the determination of the absolute number of glycogen particles in cells [5]. Using electron microscopy, the authors showed that 1 mL of rat liver contains 49 × 10^12^ growing α-particles. There are no data on the number of β-particles of glycogen in cells and their dynamics during glycogenesis.

Thus, the aim of this work was to determine the absolute number of glycogen β-particles in rat hepatocytes during glycogenesis.

## 2. Results

### 2.1. The Concentration of Glycogen in the Liver and the Content of Glycogen in Hepatocytes

**Figure 1:**
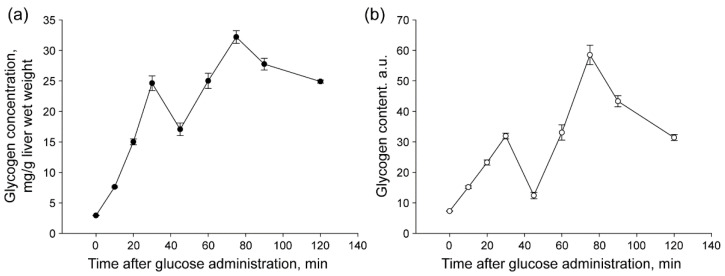
Dynamics of the liver glycogen concentration (**a**) and glycogen content in individual hepatocytes (**b**) at different stages of rat refeeding with glucose.

The results of the biochemical analysis of the glycogen concentration in the liver of fasted rats and after administration of glucose to animals are shown in the Figure 1a. The concentration of glycogen in the liver of rats after 48 h of fasting was at a low level of 2.94 ± 0.04 mg/g of wet liver weight. Administration of glucose to starved animals initiates the synthesis of glycogen, which leads to a rapid increase in its concentration in the liver. After 10 min, the concentration of glycogen in the liver increased to 7.63 ± 0.15 mg/g (*p* < 0.001). Subsequently, the synthesis of glycogen, which occurred at different rates, led to an even greater increase in the concentration of glycogen in the liver. At 75 min after the start of refeeding, there was an 11-fold increase in the concentration of glycogen in the liver compared to its concentration in fasted rats (Figure 1a).

Cytofluorometry of glycogen in individual hepatocytes showed that changes in its content during refeeding after *per os* administration of glucose to fasting rats are similar to the result of biochemical analysis (Figure 1b). The correlation coefficient between the data of biochemical and cytochemical analysis was 0.905 (*p* < 0.05), which indicates a high preservation of glycogen in isolated hepatocytes.

### 2.2. The Absolute Amount of Glycogen in Individual Hepatocytes

**Figure 2:**
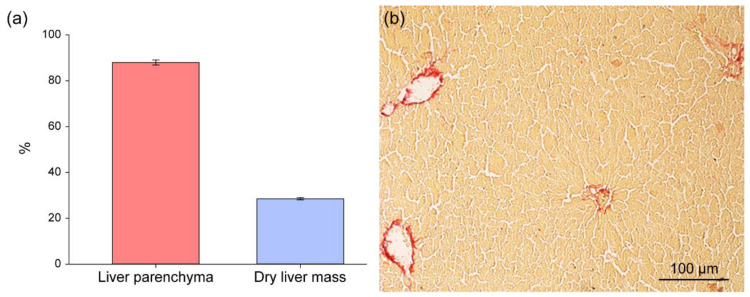
A percentage of the parenchyma and dry mass of rat liver (**a**). Rat liver section stained with picrosirius for morphometric analysis of liver parenchyma (**b**).

The parenchyma in the liver of rats, according to our data, occupies 88.0 ± 1.1% of the volume, and the dry weight of the liver is 28.5 ± 0.5% of its wet weight (Figure 2), i.e., the conversion factor from wet to dry liver mass (f) is 0.285. Therefore, the dry weight of the parenchyma in 1 g of wet liver is 250.8 ± 5.2 mg. It has been shown that fasting does not affect the percentage of liver dry mass (conversion factor value) and it remains unchanged during fasting [21].

According to our data, the dry weight of hepatocytes (DWH) in fasting rats, on average, was 621 ± 2 pg, and the number of parenchyma cells in 1 g of wet liver was 4.04 ± 0.08 × 10^8^. Administration of glucose to animals increased DWH, which at 2 h after the start of refeeding was 810.4 ± 3.3 pg (Table 1). An increase in DWH, of course, leads to a decrease in the number of hepatocytes in 1 g of the liver.

The obtained data on the number of cells and the concentration of glycogen in 1 g of raw liver (Figure 1a) allow us to calculate the absolute content of glycogen in the hepatocyte. One hepatocyte in fasting rats contains, on average, 7.28 ± 0.17 pg of glycogen. During glucose refeeding of rats, glycogen content increases rapidly, reaching its maximum value after 75 min (Table 1).

### 2.3. Characterization of β-Particles

Using the method described in detail in the article by Bezborodkina et al., 2021 [18], the number of glucose residues in β-particles was determined at different stages of glycogen accumulation after administration of glucose to starved rats. Briefly, the technique is based on:

(1) Detection of cells with the lowest glycogen content in the population of hepatocytes;
(2) The assumption that glucose residues on the outer tiers of β-particles are associated with key enzyme proteins directly involved in glycogenesis and glycogenolysis, and residues located on the inner tiers of β-particles are free from proteins;
(3) The assumption that glycogen synthase, glycogen phosphorylase and the debranching enzyme make up the majority of the proteins of the β-particle and, in accordance with their diameter, are associated with 7, 9 and 9 glucose residues.

Thus, we found that in fasted rats, the average number of glucose residues in the β-particle was 172 ± 4 and it significantly increased during 2 h of refeeding (Table 2).

Using the theoretical concepts of the distribution of the number of glucose residues on the tiers of β-particles, which were counted by the formula 13 × 2^n−1^, where n is the number of the tier, and the dependence of the particle diameter on the number of glucose residues on the tiers [15,20], our data made it possible to determine the number of tiers occupied by glucose residues in particles, the number of residues on each tier of particles, and thus calculate the diameter, volume of particles and their mass (Table 2).

### 2.4. Absolute Number of β-Particles in Hepatocytes

**Figure 3:**
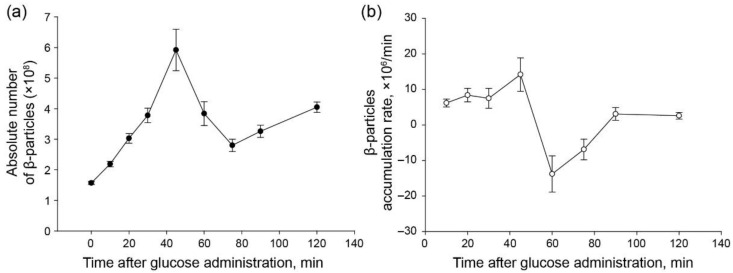
Dynamics of the absolute number of β-particles in hepatocytes (**a**) and their accumulation rate (**b**) at different time intervals after glucose administration to fasting rats.

Data on the mass of the β-particle (Table 2) and the content of glycogen in the hepatocyte (Figure 1b) allow us to determine the number of particles, on average, per hepatocyte during glycogenesis. In fasted rats, the number of particles in an individual cell was 1.57 ± 0.05 × 10^8^. The number of particles increased during the first 45 min of refeeding, after which it decreased sharply and increased again by 120 min after administration of glucose to starved animals (Figure 3a). Our data indicate that the formation of glycogen particles appears to begin immediately after the glucose administration to the animals and proceed at a rate of 6–8 × 10^6^/min (Figure 3b).

## 3. Discussion

Fasting, even if not too long, leads to significant changes in the body. Body weight of rats during 48 h of fasting decreased, according to our data, by 20.8% (*p* < 0.001), and liver weight decreased by 27.7% (*p* < 0.001). A significant part of the energy necessary for the life of cells is released as a result of β-oxidation of fatty acids. Gluconeogenesis and ketogenesis become the main processes in the liver. Glycogen stores in the liver are severely depleted [22,23,24,25].

Determination of the concentration of glycogen in the rat liver after 48 h of fasting showed that it was at a very low level: 2.94 ± 0.04 mg/g (14.7 μM/g) of the wet weight of the liver (Figure 1a). Similar data for the concentration of glycogen in the liver after 48 h of fasting (4–23 µM/g of wet weight of the liver) were obtained in other works [23,26,27,28,29].

Foods, especially those high in carbohydrates, contribute to the massive deposition of glycogen in the liver. At the end of the absorption phase after the administration of glucose to fasted animals, the glycogen concentration can reach more than 5% of the raw weight of this organ [30,31,32,33]. The administration of glucose to fasted animals stimulated intensive glycogen synthesis, which led to a rapid increase in its concentration in the liver. After 10 min, the concentration of glycogen in the liver increased by 2.6 times (up to 7.63 ± 0.15 mg/g, *p* < 0.001). Subsequently, the synthesis of glycogen, which occurred at different rates, led to an even greater increase in the glycogen concentration in the liver (Figure 1a). Cytofluorometry of glycogen in individual hepatocytes showed that changes in its content during refeeding after *per os* administration of glucose to fasted rats are similar to the data of biochemical analysis (Figure 1b).

Although our work was devoted only to the first half of the absorption phase, the total duration of which is ~4 h [34,35], the concentration of glycogen in the liver after glucose administration in fasted rats increased by more than an order in 2 h (Figure 1a). It should also be noted that the rate of glycogenesis during the studied time interval was not constant: periods of rapid glycogen synthesis (0–30; 45–75 min) alternated with periods when glycogen degradation prevailed over its synthesis (30–45 min; 75–120 min) (Figure 1a,b).

The data presented in (Table 1) indicate that the administration of glucose is accompanied not only by an increased deposition of glycogen in hepatocytes, but also by an increase in their dry mass. The DMH increased by 11.6% (*p* < 0.001) in 10 min after glucose administration, and by about 30% after 120 min as compared with the DMH of fasted rats. However, it should be noted that the increase in DMH during glycogenesis is not associated with the accumulation of glycogen in cells, since the value of the specific increment in the refractive index α for carbohydrates during dry cell mass interferometry differs significantly from that for proteins [36]. Our calculations showed that even if carbohydrates make up 20% of the total DMH, their contribution to the dry mass of cells will not exceed 3.2%. Therefore, the influence of glycogen content in cells on the change in their dry mass during refeeding can be neglected. Based on the fact that DMH is more than 80% accounted for by the protein content [37], we may conclude that glucose causes not only rapid glycogen synthesis, but also protein accumulation in the hepatocytes of fasted rats. An increase in the content of proteins in hepatocytes after the administration of pure glucose to fasted rats occurs, apparently, due to a partial redirection of the flow of amino acids appearing in the liver after gluconeogenesis to protein synthesis in hepatocytes [38,39].

The content of glycogen in cells can change both by changing the number of β-particles and by increasing or decreasing the number of glucose residues in them. Determination of the number of β-particles in hepatocytes showed that the cells of fasted rats contain ~1.6 × 10^8^ glycogen particles. During refeeding of rats with glucose, the number of β-particles gradually increases and reaches a maximum (~5.9 × 10^8^) in 45 min after *per os* administration of glucose to fasted animals, but then rapidly decreases and then changes insignificantly (Figure 3a). The data obtained indicate that the amount of β-particles of glycogen in hepatocytes is not constant. In cells, apparently, there is a continuous synthesis and degradation of glycogen particles, and at different stages of life, one or another process predominates. An analysis of the dynamics of the number of β-particles after glucose administration in starved animals (Figure 3) suggests that during refeeding, not only the synthesis of additional glycogen molecules occurs, but the replacement of “old” molecules by “new” ones. We believe that the number of old glycogen particles (~1.6 × 10^8^) does not change until about 45 min, but then they degrade and disappear completely by about 75 min after the start of refeeding.

De novo formation of glycogen particles appears to begin immediately after the glucose administration to the animals and proceed at a rate of 6-8 × 10^6^/min (Figure 3b). Gradually increasing, the number of de novo particles reaches a maximum (~4.3 × 10^8^) by 45 min after the start of refeeding (Figure 3a). Then, within half an hour (from the 45th to 75th min), the “old” β-particles and, possibly, a small part of them, formed within 60 min of refeeding, are destroyed. As a result, 2 h after the start of refeeding, the number of de novo formed glycogen particles exceeded the initial level by about two times.

There are no data on the number of β-particles in cells. However, one work is known [5] devoted to the determination of the absolute number of glycogen α-particles in rat hepatocytes. Using a mathematical model, the authors found that in 1 mL of liver with low glycogen concentration, the number of growing α-particles associated with glycogen synthase was 49 × 10^12^. In addition, the authors showed that a 7-fold difference in glycogen content was accompanied by an only 1,8-fold increase in the average volume of particles with an increase in their number by 4 times. In contrast to the data of Devos et al., 1983 [5], on α-particles of glycogen, the results of our study indicate an inverse relationship between the volume of β-particles and their number. During glycogenesis, when the average volume of β-particles increases by about eight times, the number of particles increases only by about two times (Table 2).

Theoretically, a mature β-particle includes ~55,000 glucose residues arranged in 12 tiers [10,11,12]. Of course, it cannot be ruled out that such completely formed particles may appear among hundreds of millions of glycogen particles in cells. However, in reality, the presence of 12-tier glycogen molecules seems to be an extremely rare event. Determination of the amount of glucose residues in β-particles in fasting rats and then during refeeding showed that particles in hepatocytes contain many times less glucose residues than a mature (completely formed) β-particle (Table 2). Among the large number of measured hepatocytes at different stages of refeeding, the cell with the highest glycogen content had β-particles, the number of glucose residues in which, on average, was only 7767. This number of glucose residues is about seven times less than in a mature β-particle, and its diameter corresponded to 30.6 nm.

Most studies of the size of β-particles have been carried out with the help of electron microscopy. They showed that the diameter of glycogen granules fluctuates in the range of 20-30 nm [40,41,42,43,44]. In a study of glycogen synthesis in skeletal muscles, the diameter of β-particles increases by about 4 nm (on average from 24.9 nm to 28 nm), while in the course of glycogenolysis it decreases to 24.4 nm [41]. After intensive and prolonged physical exercise, the diameter of glycogen particles in skeletal muscles gradually increases throughout a 48-h-long recovery period from 13.3 nm to 23.8 nm [45]. Assuming that the diameter of a glycogen molecule increases with each additional tier ~by about 3.8 nm [20], our data indicate that the average diameter of glycogen molecules in hepatocytes of starved rats would be ~10.8 nm, and in hepatocytes of rats 75 min after refeeding it would be ~21.8 nm.

The size of glycogen molecules as calculated in our study (Table 2) is somewhat smaller than the values given in electron microscopy works. However, structures usually considered as glycogen particles under an electron microscope are in fact glycosomes: organelles consisting of protein and polysaccharide [46]. In addition, the size of glycogen particles is thought to increase after their association with various enzymes [42]. We determined the size (diameter) of the glycogen molecules based only on the size of the polysaccharide part, ignoring the protein cap at the molecule’s surface, which consists of various enzymes of glycogen metabolism. This explains a somewhat smaller size of glycogen molecules in our study as compared to electron microscopy studies.

It has been shown that despite the significant variability in the diameter of β-particles in cells, its value according to many electron microscopic studies is ~25 nm, which corresponds to 7–8-tiered glycogen particles [15,43]. Such particles contain 2.0–3.5 × 10^3^ glucose residues, which corresponds to only 6–7% of the number of residues in a fully formed β-particle [10,11,12]. The reasons why hepatocytes use such a small fraction of their theoretical glycogen storage capacity remain unknown. In some pathologies in humans and animals (for example, glycogen storage disease type 1a, liver cirrhosis), associated with a strong weakening of the activity of glucose-6-phosphatase or its complete absence in the liver, a large amount of glycogen accumulates in hepatocytes. The glycogen content in cells can exceed the regular level several times [18,47,48]. However, it remains unclear whether the accumulation of glycogen in these diseases occurs due to an increase in the number of β-particles in hepatocytes or due to an increase in the number of glucose residues in the particles (filling new tiers), and, accordingly, an increase in their size.

Thus, administration of glucose to rats fasted for 48 h stimulates intensive glycogenogenesis in hepatocytes. The synthesis of glycogen was accompanied by the formation of new β-particles in hepatocytes. The absolute number of β-particles, which was ~1.6 × 10^8^ in the hepatocytes of fasting rats, approximately doubled during an hour of glucose refeeding in rats. Despite the increase in the number of β-particles during glycogenesis, the main contribution to the deposition of glycogen in hepatocytes is made by the process of increasing glucose residues in β-particles, which is accompanied by an increase in particle size. The average diameter of glycogen β-particles, which was about 11 nm in hepatocytes of starved rats, increased during glycogenesis to about 21 nm, which corresponds to particles with 6–7 tiers of glucose residues.

## 4. Materials and Methods

### 4.1. Animals and Procedures

We used 27 outbred adult male white rats aged 10 months with body masses of 300–350 g obtained from Rappolovo laboratory animal nursery (Leningrad region, Russia), quarantined for 2 weeks, then housed in a standard animal facility and fed a complete compound feed (Laboratorkorm, Moscow, Russia). At the beginning of the experiment, the rats were fasted for 48 h (water ad libitum) for depletion of glycogen reserves in the liver and then given 30% glucose solution orally (4g/kg of body mass) once. The rats were sacrificed by decapitation immediately after the termination of fasting (0 min) and 10, 20, 30, 45, 60, 75, 90 and 120 min after the administration of glucose. All animals before decapitation were pre-anesthetized with hexobarbital sodium (60 mg/kg). Three rats were used for each time point. Pieces of their liver were used for histological, biochemical and cytochemical research.

The study was carried out in accordance with the principles of the Helsinki Declaration, Order of the Ministry of Health of the Russian Federation No. 199n (1 April 2016) “On the approval of the rules of good laboratory practice”, and the recommendations of the Bioethics Committee of Saint Petersburg State Chemical and Pharmaceutical University of the Ministry of Health of the Russian Federation.

### 4.2. Determination of the Transition Coefficient from the Wet Weight of the Liver to the Dry (f)

Small pieces (ca. 1 × 1 × 1 cm, *n* = 15) were cut out from the rat liver immediately after isolation and were quickly weighed. Then, the pieces were placed in a thermostat and dried at t° = 60 °C to constant weight. The constant weight of the pieces was established within approximately 1 week.

### 4.3. Tissue Preparation

Pieces of rat liver were fixed in 10% neutral formaldehyde and embedded in paraffin blocks by the routine method. Then, blocks were cut with the Reichert microtome (C. Reichert, Wien, Austria) into sections approximately 5–6 μm thick. To reveal the connective tissue, the sections were stained with picrosirius red (0.01% solution of Sirius red F3BA (Bio-Optica Milano SPA, Italy).

### 4.4. Histological Analysis

A percentage of the parenchyma (R) in the sections were assessed by an AxioVert 200M microscope (Carl Zeiss, Jena, Germany) equipped with a 10×/0.30 objective and a digital camera Leica DFC420C (Leica Microsystems, Wetzlar, Germany). For each animal, 20–30 fields of vision were analyzed using the ImageJ software (National Institutes of Health, Bethesda, Maryland, USA, https://imagej.nih.gov/ij, accessed on 1 July 2022) [18].

### 4.5. Isolated Hepatocyte Smears on Object Slides

A piece of rat liver (ca. 2 × 2 × 2 mm) was placed in phosphate buffer I, pH 8.0 (475 mL 0.066 M Na_2_HPO_4_*2H_2_O, 25 mL 0.067 M KH_2_PO_4_, 500 mL 0.15 M sucrose) for 5–10 min, t = 20–22 °C, and then in phosphate buffer II, pH 7.4 (400 mL Na_2_HPO_4_*2H_2_O, 100 mL KH_2_PO_4_), at the same temperature for 10–15 min. A suspension of cells was obtained by gently stirring the piece in a drop of buffer II with pincers. The suspension was smeared on the surface of the object slide using quartz glass with a polished edge. The preparations were fixed in 100% methanol and air-dried. These conditions provide high glycogen preservation in isolated hepatocytes.

### 4.6. Determination of the Dry Weight of Hepatocytes (DWH)

DWH was measured with the help of an interference microscope MBIN-4 (LOMO, St. Petersburg, Russia) with a 10×/0.30 objective and light interference filter with λmax = 550 nm. We equipped this microscope with a CCD camera connected to a computer [39]. The measurements were carried out in two steps. First, the optical path difference for the hepatocyte and the embedding medium (glycerin in our case) was measured. Then, using the ImageJ software (National Institutes of Health, Bethesda, MD, USA, https://imagej.nih.gov/ij, accessed on 1 July 2022), the area of the cell was measured (in μm^2^). DWH was calculated according to the formula:

P = (δ∙× S)/(100 × α),

where P is the dry weight of the cell (in picograms, 10^−12^ g) and δ is the path-length difference (in cm); δ was determined according to the formula: δ = (φ1 − φ2)∙λ/K, where φ1 and φ2 are readings of the Senarmont compensator scale, λ is the length of the light wave (546 nm), K = 1800, S is the cell area (in cm^2^) and α is the specific increment of the refractive index, making up 0.00095 cm^3^/g for proteins in glycerin [36,37].

### 4.7. Calculation of The Hepatocyte Number in The Liver of Rats

The number of hepatocytes in the liver of rats was determined by the formula:

N = P × R × f/M,

where N is the number of hepatocytes in the liver; P is the wet weight of the liver, g; R is the percentage of parenchyma in the liver; f is the coefficient of transition from the wet liver weight to dry; M is the average dry weight of one hepatocyte, g.

### 4.8. Determination of Glycogen Concentration in Rat Liver

Liver samples were weighed and digested in 1 mL of hot 30% KOH (for 60 min at 100 °C), and after that 70% ethanol was added to precipitate the glycogen from the alkaline digests. The samples were centrifuged for 30 min at 1000 g, washed successively with 80% and 96% ethanol, and then centrifuged again. The pellets were hydrolyzed in 3 mL of 2N H_2_SO_4_ for 2.5 h in a boiling water bath. The hydrolysate was neutralized with 5N NaOH up to pH 7.8–8.0. The amount of the glucose formed was determined by the glucose oxidase method. The results were expressed as micromole glucosyl units per g wet weight of the liver.

### 4.9. Determination of Glycogen Content in Hepatocytes

The preparations were stained for glycogen with the help of the fluorescent variant of Periodic acid–Schiff (PAS) reaction, with auramine-SO_2_ (Au-SO_2_) used as Schiff’s reagent. Smears of isolated hepatocytes on object slides were oxidized in periodic acid (0.8% KIO_4_ solution on 0.23% HNO_3_) for 90 min. Then, preparations were washed in running water for 5 min and transferred into distilled water. They were taken out of distilled water and placed into a 0.3% SO_2_-saturated auramine solution (0.2 mL thionyl chloride per 100 mL of the dye) for 90 min at room temperature. After staining, preparations were removed from the staining solutions, rinsed thrice in distilled water and thrice in sulfur water (5 g K_2_S_2_O_5_, 950 mL of water, 50 mL of 1 N HCl), for 3 min each time, in order to remove non-specifically bound stain. Then, the preparations were washed under running water for 20 min, rinsed in distilled water and placed into an ascending alcohol series (70%, 96% and 100%) for 5 min in each alcohol. After that, the preparations were air-dried and stored in the dark. Directly before measurements the stained preparations were embedded into non-fluorescent paraffin oil (JSC REACTIV, St. Petersburg, Russia).

Fluorescent images of Au-SO_2_-stained hepatocytes were obtained with the use of an Axioskop microscope (Carl Zeiss, Jena, Germany) equipped with a Plan-NEOFLUAR 20x/0.50 objective, Filter Set 10 and a digital monochrome high-sensitivity CCD camera Leica DFC360 FX (Leica Microsystems, Wetzlar, Germany). Fluorescence intensity of 150–200 cells obtained from each animal was measured with the use of the ImageJ software (National Institutes of Health, Bethesda, Maryland, USA, https://imagej.nih.gov/ij, accessd on 1 July 2022).

### 4.10. Statistics

The results were processed statistically using Sigma Plot for Windows 11.0 standard software package (Systat Software Inc., Chicago, IL, USA). The data were given as mean ± standard error of the mean and as a weighted mean and its error. Differences between the mean values were detected using Student’s t-criterion.

## Figures and Tables

**Table 1:** Average dry weight of hepatocyte and absolute glycogen content in them at different time intervals after glucose administration to fasting rats.

Time, min	Dry Weight of Hepatocyte, pg	Glycogen Content Per Hepatocyte, pg
0	621 ± 2 ^1^	7.28 ± 0.17
10	693.2 ± 3.1	21.09 ± 0.62
20	744.2 ± 3.2	44.54 ± 1.70
30	778.7 ± 3.2	76.49 ± 4.08
45	800.4 ± 3.2	54.54 ± 3.51
60	806.3 ± 3.3	80.42 ± 4.40
75	808.3 ± 3.3	103.87 ± 3.90
90	809.8 ± 3.3	89.52 ± 3.51
120	810.4 ± 3.3	80.58 ± 1.77

^1^ Here and below, the data are given as mean ± SE, *n* = 3.

**Table 2:** Parameters of β-particles at different time intervals after glucose administration to fasting rats.

Time, min	Number of Glucose Residues in β-Particle	Mass of β-Particle, ^1^ pg × 10^−8^	Diameter of β-Particle, nm	Volume of β-Particle, nm^3^
0	172 ± 4	4.63 ± 0.12	10.8	659.3
10	357 ± 10	9.61 ± 0.30	14.6	1628.8
20	547 ± 19	14.72 ± 0.57	16.7	2437.6
30	751 ± 22	20.21 ± 0.66	18.6	3367.8
45	342 ± 29	9.21 ± 0.87	14.3	1530.5
60	778 ± 59	20.94 ± 1.77	18.8	3477.7
75	1377 ± 75	37.06 ± 2.25	21.8	5422.3
90	1019 ± 43	27.42 ± 1.32	20.1	4250.1
120	739 ± 23	19.89 ± 0.69	18.5	3313.8

^1^ It is known that the mass of one glucose molecule is 2.99 × 10^−10^ pg, and one molecule of water is 0.299 × 10^−10^ pg. Consequently, in the hepatocyte of fasted rats, the mass of one β-particle containing 172 glucose residues will be equal to 4.63 ± 0.12 × 10^−8^ pg, and after 120 min it will reach 19.89 ± 0.69 × 10^−8^ pg.
